# Ultrasound evaluation of intraluminal magnets in an ex vivo model

**DOI:** 10.1007/s10140-023-02160-7

**Published:** 2023-07-22

**Authors:** Jason J. Lee, Amanda L. Rugg, Crystal K. Wu, Garrett J. Hamblin, Michael C. Larson

**Affiliations:** 1 Department of Medical Imaging, University of Arizona College of Medicine, 1501 N. Campbell Ave, P.O. Box 245067, Tucson, AZ 85724 USA; 2 Department of Pediatrics, Tucson Medical Center, 5301 E. Grant Road, Tucson, AZ 85712 USA; 3 LewisGale Medical Center, 1900 Electric Rd, Salem, VA 24153 USA; 4 Department of Radiology, University of California – Davis Health, 4860 Y Street, Ste 3100, Sacramento, CA 95817 USA

**Keywords:** Ultrasound, Foreign body, Magnet ingestion, GI imaging, Radiography, Risk stratification

## Abstract

**Purpose:**

The management of foreign body ingestion proves to be a challenge. Magnets pose a unique set of risks when ingested due to their attractive forces and subsequent risk of adherence, pressure necrosis, and perforation complications. Radiographs only provide a limited snapshot in the setting of multiple magnet ingestion when the risk of complication is highest. We hypothesize that abdominal ultrasound (US) has the potential to supplement radiographs in assessing ingested magnets by determining the presence of bowel loop entrapment and of any extraluminal fluid.

**Methods:**

We recreated various scenarios of magnet configurations using animal cadaveric bowel models. X-ray and US images were obtained in various bowel-magnet orientations.

**Results:**

We identified several key US features to suggest bowel wall tethering. These include direct visualization of bowel wall entrapment between magnets (what we term the “dangerous V sign”), anti-dependent positions of the magnets, and inability to separate loops of bowel with compression.

**Conclusion:**

These findings could potentially provide valuable information when directing the urgency of intervention in foreign body ingestion. Ultrasound may supplement and improve the current guidelines in management of magnet ingestion.

## Introduction

It is well known that young children often put objects in their mouths, an action driven by their curiosity to interact with the world. Accidental ingestion of non-food items is common, occurring mostly in children between the ages of 6 months and 3 years [1] while 75% of cases occur in children under the age of five [2]. Children with developmental delay or behavioral problems are particularly at risk for multiple ingestions [3].

Magnets are one of the most commonly ingested foreign bodies [4] that result in thousands of emergency department visits annually and even more radiology cases over the past few decades [5, 6]. Novel products such as shiny magnets are attractive to young children and come in various shapes (including circular, square, and tablets) and small sizes that are easily swallowed, leading to products being withdrawn/recalled [6]. Rare earth magnets, most commonly made from neodymium, are at least 5 to 10 times more powerful than traditional ferromagnets and are marketed as desk toys and stress relievers that can still be easily purchased from the internet [7]. Given the availability of these objects, the incidence of magnet ingestions of late has increased at an alarming rate [5, 8]. These rare earth magnets accounted for 16,386 cases of emergency department visits over a 10-year period in the United States and are a dangerous cause of foreign body ingestion-related morbidity and mortality [9].

Many swallowed foreign bodies can be expected to pass through the gastrointestinal (GI) tract uneventfully [6]. However, ingestion of more than one magnet can lead to entrapment of the GI tract from attractive magnetic forces. The resulting adhesion and pressure injuries can result in perforation, fistulae, obstruction, or infections that result in serious consequences. The North American Society of Pediatric Gastroenterology, Hepatology, and Nutrition (NASPGHAN) has provided guidance on the management of ingested magnets in children [7].

**Fig. 1:**
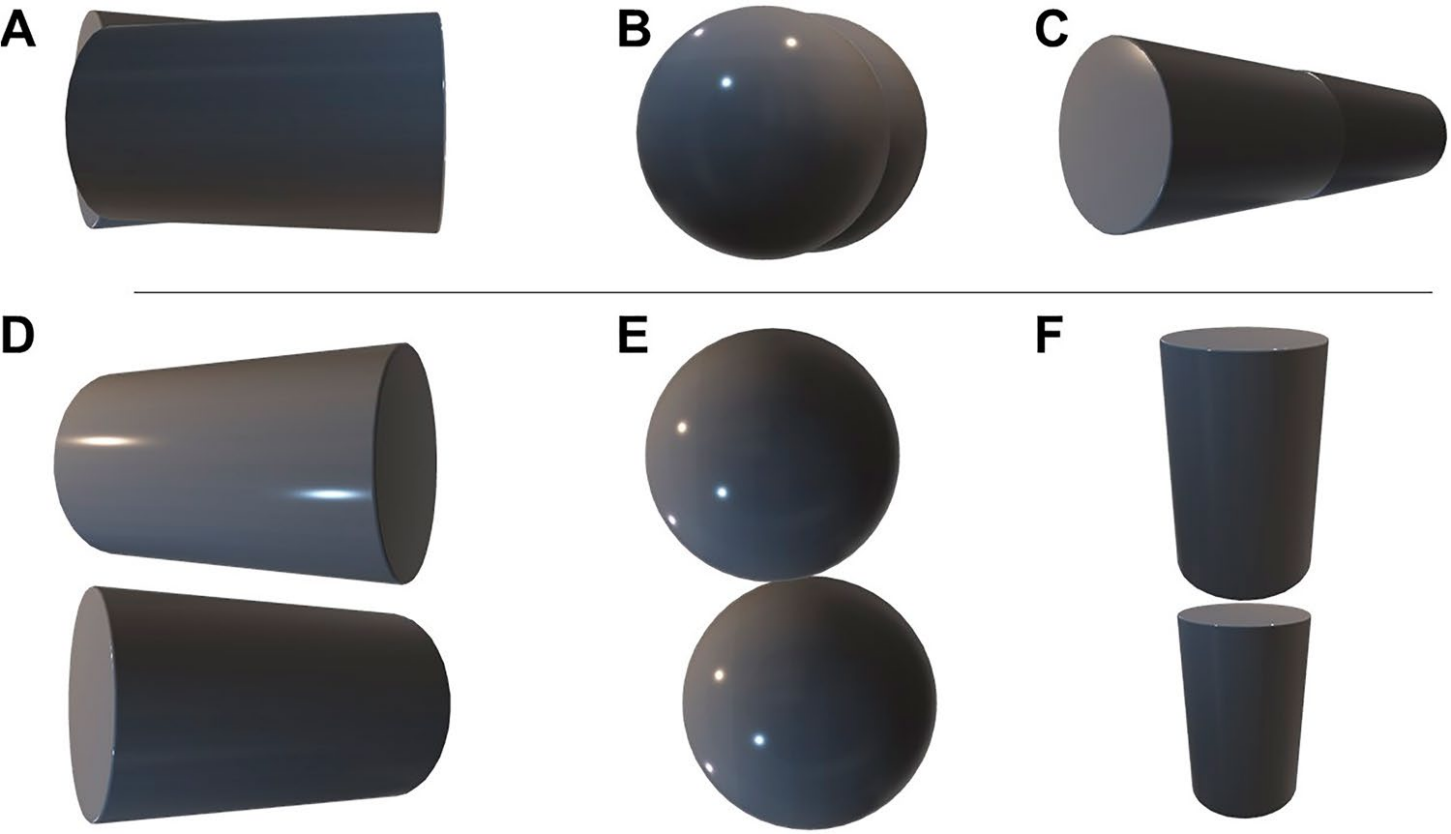
3D models highlighting limits to projection radiography. **A**, **B**, **C** 3D objects shaped like common household magnets are oriented such that a single projection would suggest to the viewer that there is only one object present. **D**, **E**, **F** Orthogonal views of the 3D objects clearly demonstrate 2 distinct objects are present

Once magnet (or other metallic foreign body) ingestion is confirmed by abdominal radiograph, ascertaining single versus multiple magnet ingestion is key in deciding subsequent management. A single swallowed magnet can be managed conservatively with appropriate education of the parents and child. Multiple magnet ingestions or co-ingestion of a single magnet with another metallic object should be treated with increased urgency because of the high risk of complications such as obstruction, perforation, and/or infection. The present guideline recommends obtaining a lateral abdominal radiograph if magnets are present on initial flat plate film [7]. However, practical limitations of radiographs arise when multiple magnets adhere together and partially overlap on a single view or shift location with changing patient positioning between orthogonal views leading to potential misdiagnosis and the inability to assess bowel loop entrapment between multiple magnets [10, 11]. The limitations of standard projection radiographs can be understood with the aid of Fig. 1, showing 3D models overlapping from one view that could be confused for a single object, but clearly representing 2 objects when visualized from an orthogonal view. Depending on the proximity of the objects and overlap, discriminating one from multiple objects on a single projection can be impossible.

The goal of this ex vivo pilot study is to assess the utility of ultrasound in the evaluation and risk stratification of magnet ingestion. We hypothesize that abdominal ultrasound has the potential to be a critical adjunct to radiographs in assessing single versus multiple magnet ingestion as well as determining the presence of bowel loop entrapment between multiple magnets.

### Materials and methods

**Fig. 2:**
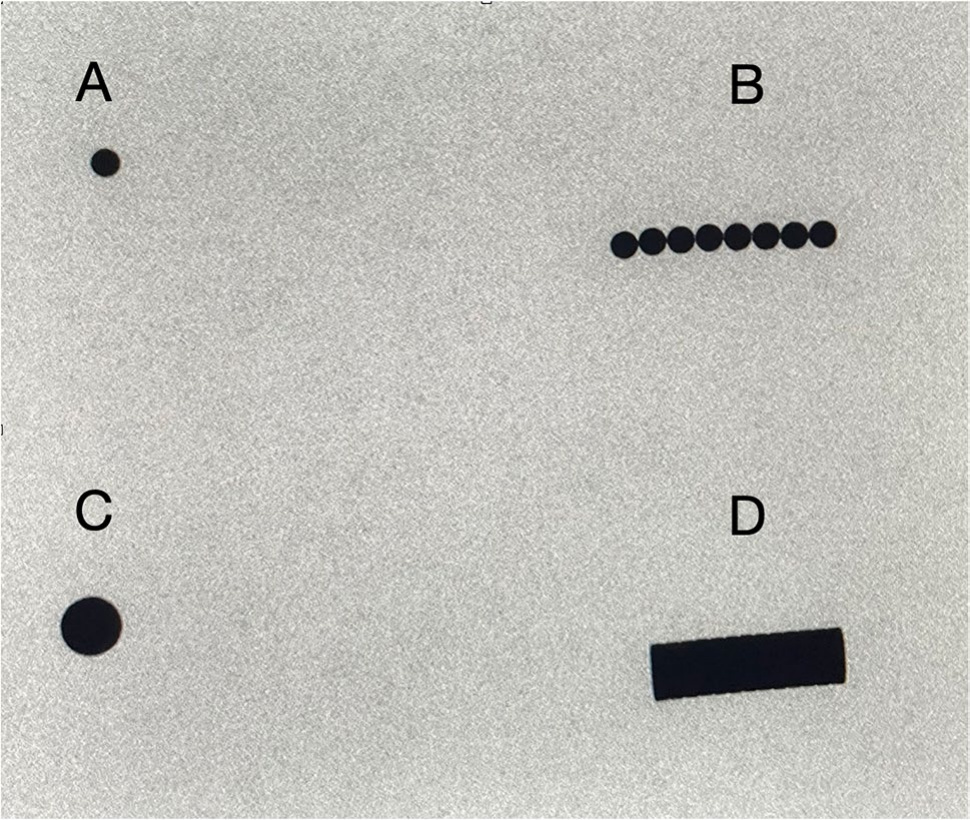
X-ray views of magnets used in this study. Fluoroscopic spot images of (**A**) single Buckyball magnet, **B** 8 Buckyball magnets aligned, **C** single button magnet, and (**D**) stack of button magnets

**Fig. 3:**
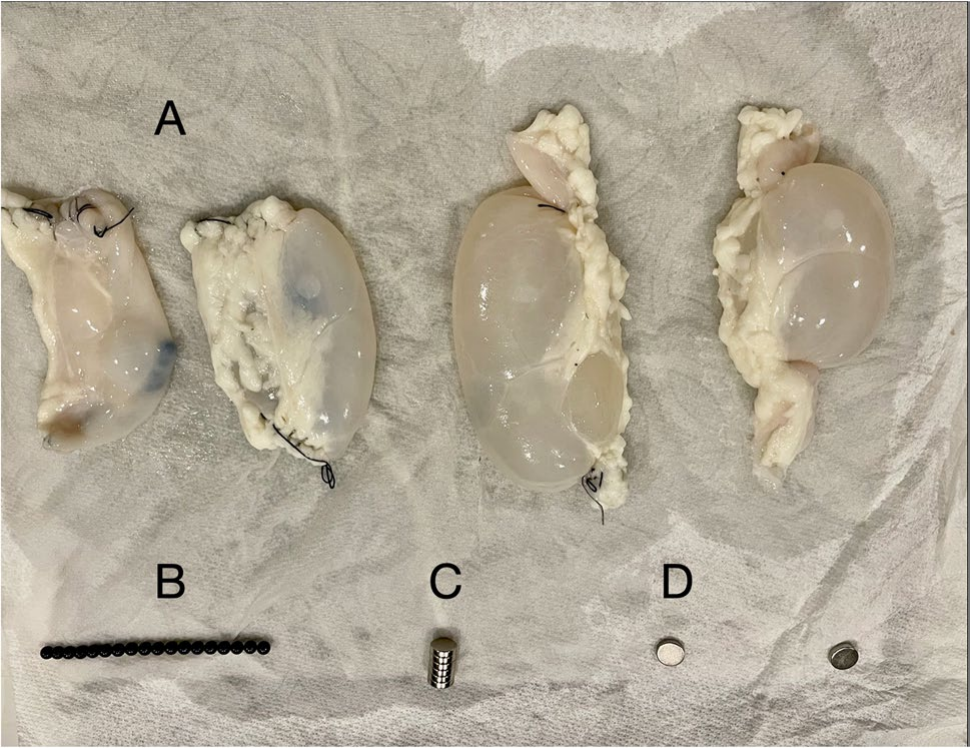
Small bowel segments used in this study. **A** Segments of bovine small intestine ligated at distal ends. **B** A string of Buckyball magnets. **C** A stack of button magnets. **D** Single button magnets

The first step in this investigation was to create a simulated environment reflecting imaging properties seen with a routine abdominal ultrasound; this includes mesenteric fat, bowel loops, and bowel content. Commercially available bovine small bowel was resected into short segments, various combinations of magnets (Fig. 2) inserted in, and ligated at each end (Fig. 3).

Two common varieties of magnets were used for this study. The first, commonly known as “Buckyballs,” are sold as a cluster formed by up to 216 individual round magnets about 5mm in diameter. While solid in singularity, they are malleable in clusters and can conform to multiple different geometric shapes. The second type of magnets were made from neodymium and are small button-shaped, closely resembling common household magnets. Buckyball-type magnets were arranged in a linear configuration while button magnets were stacked and x-ray images obtained (Fig. 2).

The bowel segments were filled with water and ligated at both ends (Fig. 3). They were then placed in a corn starch and water mixture designed to attenuate sound wave transmission, creating an environment mimicking the echogenic appearance of intrabdominal fat on sonography. Fluoroscopic spot images of the two types of magnets inside the bowel were also obtained in singular and multiples and shown in subsequent figures.

Sonographic images were obtained using a point-of-care ultrasound probe and accompanying Android tablet with VistaScan software (Emagine Solutions Technology, Tucson, AZ, USA). The images were acquired with a linear transducer set at 10MHz, image depth set at 5 cm, and focused at 1.5-cm depth. Additionally, corresponding x-ray images were obtained via fluoroscopy. Images were saved and transferred to a separate workstation for analysis.

All images were acquired by a radiology resident. Images were obtained with single or multiple bowel segments in various orientations. Several blinded trials were also conducted where the individual scanning was unaware of bowel loop and magnet configuration to serve as a control. Color and Doppler flow images were not obtained as blood flow could not be reproduced in cadaveric bowels.

## Results

Sonographic images were successfully obtained from the simulated abdominal environment. The corn starch mixture had a homogeneous echogenic texture resembling the attenuation of intra-abdominal fat. Bowel segments closely resembled physiologic fluid-filled bowel with adjacent mesenteric fat.

**Fig. 4:**
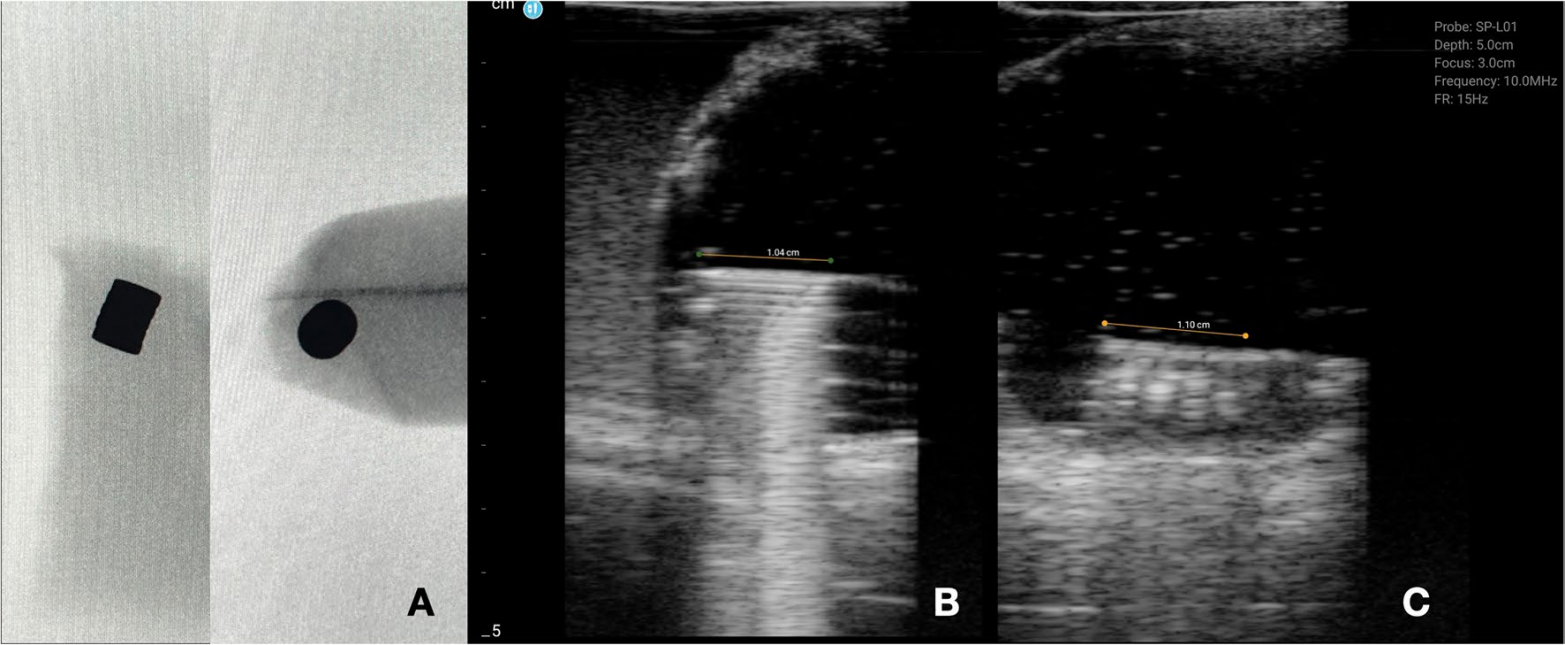
Stacked disc magnets. **A** Radiographic appearance of stacked disc magnets in longitudinal and en face configuration. **B** Transverse and (**C**) longitudinal views on sonography of the stack of magnets in horizontal orientation. Note the hypoechoic lines marking the interface of each magnet

Figure 4A shows that four disc or button magnets are aligned in a linear fashion on x-ray. However, due to variable or non-visualization of bowel loops on x-ray, the relationship of the magnets to the bowel loops is typically unknown. Figure 4B and C are a longitudinal and transverse, respectively, view of the same groups of magnets on sonography. Four semicircular echogenic objects are seen surrounded by a curvilinear structure that demonstrates classic “gut signature.” Findings are consistent with 4 button magnets within a single loop of bowel without bowel wall entrapment between the magnets; note the reverberation artifact caused by the magnets on Fig. 4B.

**Fig. 5:**
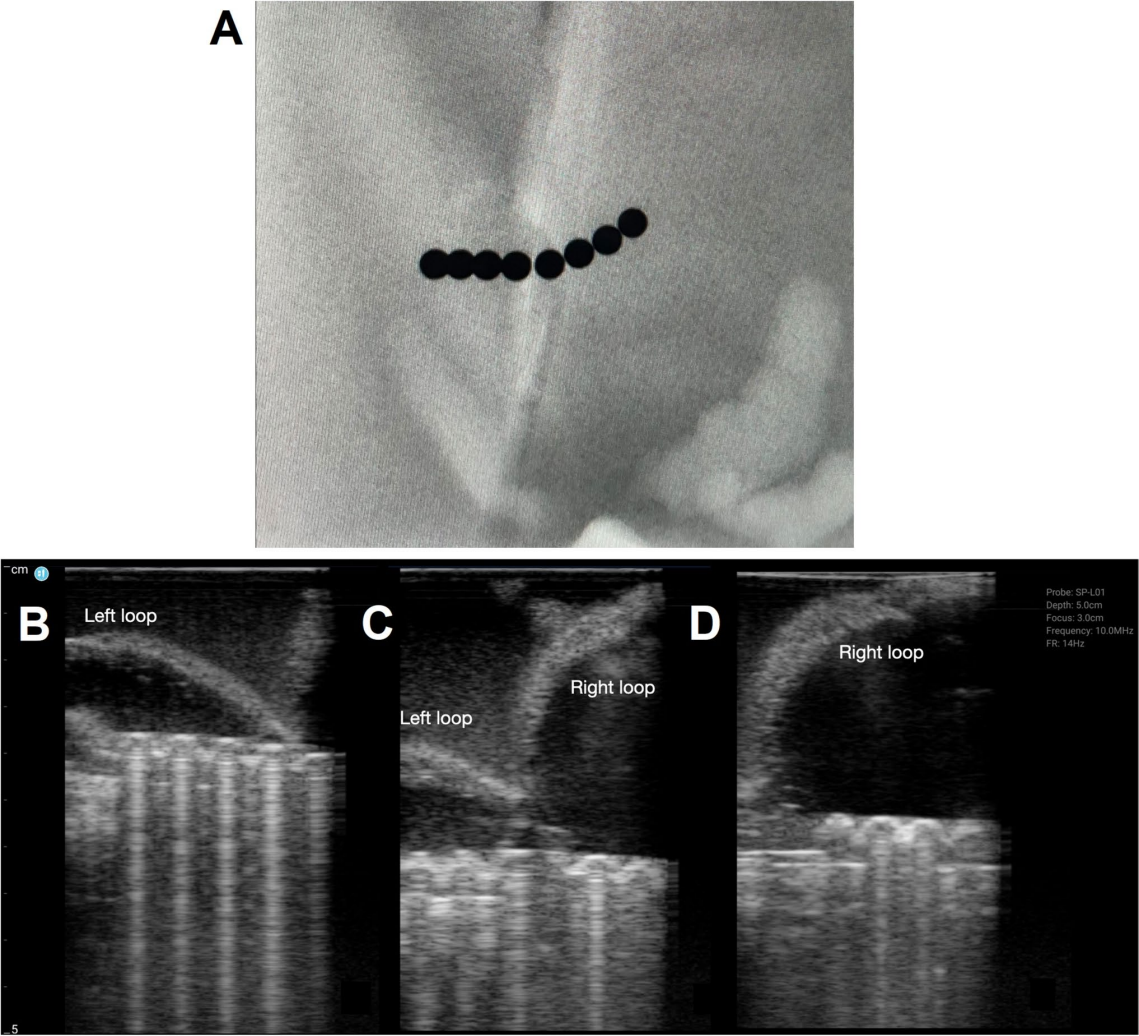
Eight Buckyball magnets in a linear orientation. **A** X-ray appearance of 8 Buckyball magnets. Four Buckyball magnets in two separate loops of bowel were tethered together by magnetic forces as seen on accompanying ultrasound images, with the (**B**) left loop, **C** both loops of bowel tethered by magnets with a “V” shape in between, and (**D**) right loop

**Fig. 6:**
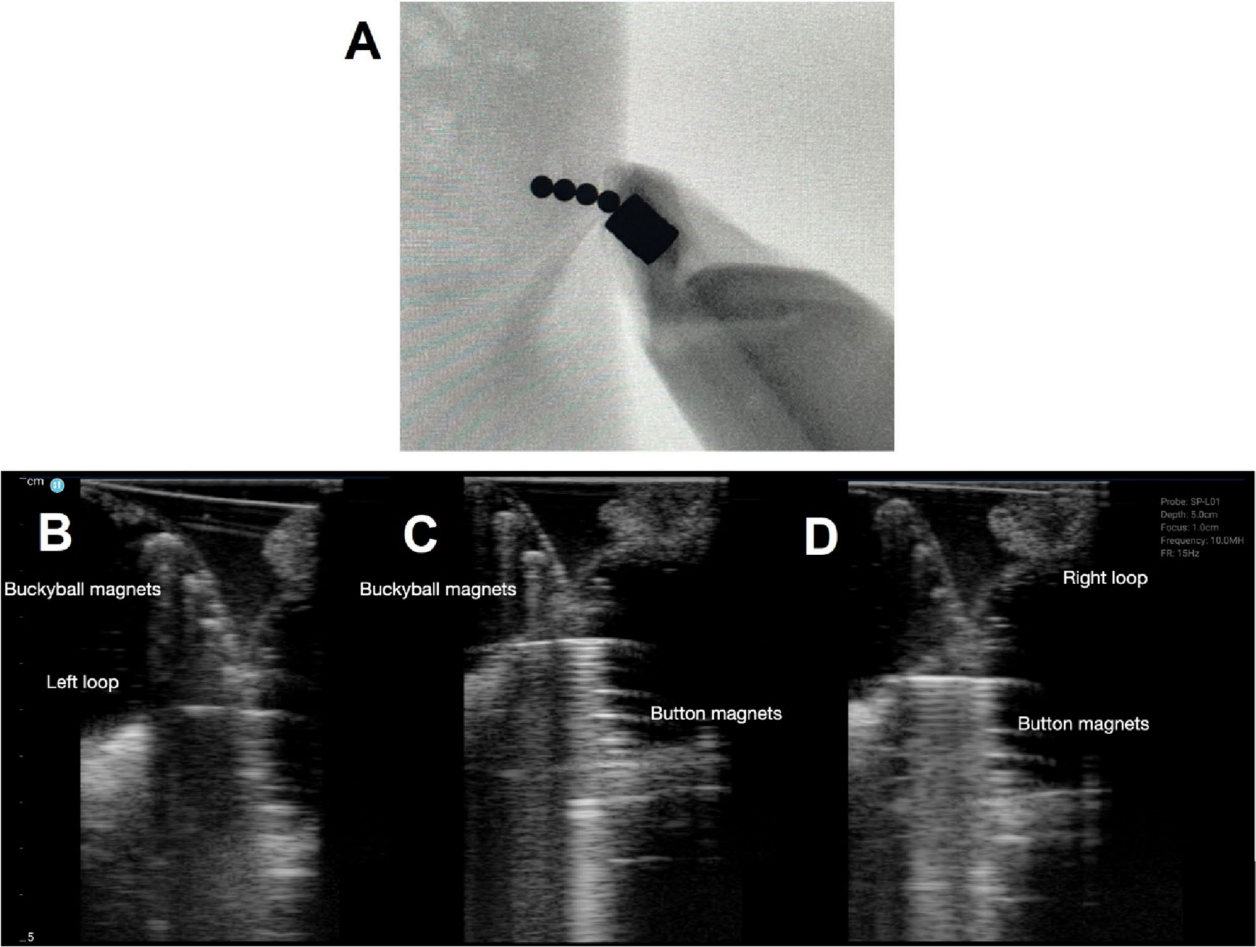
Four Buckyball magnets and a stack of button magnets in proximity. **A** X-ray appearance of the magnets in fluid in bowel. Ultrasound images of the 4 Buckyball magnets seen in the (**B**) left loop of bowel (**C**) tethered in the middle (with a “V” shape) and a (**D**) stack of button magnets seen in the right loop

Eight buckyball-type magnets in linear orientation were placed in segments of bowel and imaged as shown in Fig. 5. No information regarding bowel loops can be meaningfully interpreted due to the limitation in soft tissue discrimination by x-rays (Fig. 5A). The linear opacities seen are from plastic bags within which bowel segments were contained. Figure 6B-D show two echogenic, curvilinear structures converging at the center with magnets seen on either side. Figure 6B and D show the two loops of bowel individually. These findings are in keeping with two loops of bowel tethered together by magnetic forces. As noted by the individual scanning, the two loops were not separable with graded compression with the ultrasound probe.

Four buckyball-type magnets and a stack of button magnets were placed in proximity within the small bowel segments and imaged as shown in Fig. 6. Again, it would be difficult to postulate the relationship between magnet and bowel by x-ray alone (Fig. 6A) without the linear outlines from plastic bags as mentioned above that would not be present to aid the viewer in distinguishing adjacent bowel loops in a real clinical scenario. However, as seen in Fig. 6B-D, two bowel walls converge at the center with two different types of magnets seen in either loop. Careful manipulation of the transducer revealed four anti-dependent Buckyball-type magnets in a loop of bowel on the image left (Fig. 6B) and a stack of button magnets in another bowel loop on image right (Fig. 6D). Sonographic findings clearly demonstrate two loops of bowel tethered by two different types of magnets with bowel wall entrapment.

## Discussion

Magnets, unlike other radiolucent foreign bodies that are commonly ingested, can be easily identified on plain films. However, radiography only provides the relative location, orientation, and morphology of the object(s). The NASPGHAN guidelines recommend initial plain films to help determine the quantity of magnets ingested, but the utility of plain film ends here. In addition, radiography does not provide information on how the magnets will behave in the body. It is difficult to predict whether the magnets will progress along the GI tract uneventfully, cause an obstruction, or lead to further complications such as volvulus, bowel wall necrosis, or perforation [12, 13].

Ultrasound has been used in the past to identify the location and nature of foreign bodies in the esophagus or stomach if appropriate expertise is available. Many case reports and case series demonstrate the utility of ultrasound as an adjunct to help localize ingested foreign bodies [14–19]. Our work is novel in that we focus on the region past the pylorus while most of the foreign bodies in these previous investigations were imaged in the esophagus or stomach when still potentially retrievable by endoscopy. It is not unreasonable to suggest that abdominal ultrasound could become more frequently used in cases of magnet ingestion. It is a modality often preferred in pediatric patients for its lack of ionizing radiation exposure in the context of other gastrointestinal emergencies such as intussusception and appendicitis, and has been for decades [20–24].

Our work suggests that ultrasound offers several advantages in the scenario of magnet ingestion. First, there are distinct sonographic findings that can complement identification of the magnets seen on plain film. We have shown that both Buckyball-type and button magnets are intrinsically highly echogenic and produce significant reverberation artifact due to their smooth and highly reflective surfaces. This is congruent with findings published in a 2014 study of magnets in the stomach [25]. Furthermore, ultrasound allows us to further evaluate the orientation of magnets in relation to the bowel loops. Owing to the dynamic maneuverability of ultrasound imaging, the transducer can easily be manipulated to confirm the relationship of magnets to bowel loops, which would be impossible on conventional abdominal radiography.

Most importantly, there are several sonographic findings that can suggest the most dreaded complication: opposing bowel loops tethered together by magnetic forces. This can be directly visualized on ultrasound when two separate bowel walls converge to the center of a cluster of magnets (what we are referring to as the “dangerous V sign”), as with our findings. Magnets orienting anti-dependent to gravity is another clue, which likely means that magnetic forces from another group of magnets are holding them in place. Finally, loop tethering is suspected when bowel loops cannot be separated with application of graded compression with the ultrasound probe.

As with any imaging modality, ultrasound has limitations when evaluating bowel structures. Patient body habitus, overlying bowel loops, and presence of bowel gas can obscure the view of deeper structures or foreign bodies. These limitations are commonly encountered in abdominal ultrasound and are not specific to our application. While bowel gas may be the primary obstacle to ultrasound wave penetration, deep and directed compression allows for displacement of bowel loops and intraluminal gas [26]. Such deep compression could be more easily or accurately directed with the knowledge from a preceding radiograph of the general region of the foreign body (or bodies). We postulate a few qualities that may have the highest potential yield with ultrasound imaging. Low abdominal fat, low bowel gas, fluid-filled and dilated bowel loops, and superficial location of the affected bowel loop will all likely improve the sensitivity and image quality when evaluating magnets in the GI tract via ultrasound.

As valuable as ultrasound may be in assessing magnet ingestion, it is also important to remember that ultrasound should not be solely relied upon to make the clinical diagnosis. Ultrasound should only be used as an adjunct to (2-view) radiographs or other cross-sectional imaging to monitor disease progression and possible complications. For example, risk of bowel entrapment increases if there is space between multiple magnets on plain film [27]. In this scenario, it would be critical to conduct a thorough sweep-through to ensure that all foreign bodies seen on plain films are identified and characterized on ultrasound. (The authors recommend caution when using magnets around electronic and ultrasound equipment, as the effects of magnets on ultrasound probes is not well studied.)

Future work should include clinical studies which compare abdominal ultrasound to radiograph findings in patients who present with magnet ingestion. This would allow us to not only identify factors that would affect image quality but also to assess the reliability of identifying entrapped bowel loops in vivo. In addition, in vivo studies would make it possible to evaluate the blood supply to bowel loops with Doppler spectroscopy and changes in peristalsis pattern, which would provide additional information on potential ischemia or obstruction. This would help triage patients based on the likelihood of blood supply interruption and subsequent need for surgery. Finally, larger studies would be powered to answer questions on statistical significance and risk reduction when comparing ultrasound as an adjunct to radiograph versus radiograph alone in a head-to-head study.

## Conclusions

In conclusion, there is convincing evidence that ultrasound has the potential to be a valuable tool in assessing and managing patients with magnet ingestion, particularly in the case of multiple ingestions and bowel loop entrapment. While surgical intervention can be life-saving, surgery and anesthesia are not without costs and risks, so it is critical to appropriately diagnose and triage any complications of magnet ingestion for appropriate management. Single projection radiographs may be insufficient at detecting multiple magnets or estimating proximity of multiple objects. Ideally, ultrasound would provide insight for emergency medicine physicians and surgical specialists to quickly decide whether patients with multiple magnet ingestion require immediate surgical treatment or can be monitored conservatively.

## Data Availability

Not applicable, as no databases were created.

## References

[1] Lee JH (2018). Foreign body ingestion in children. Clin Endosc.

[2] Bronstein AC, Spyker DA, Cantilena LR, Rumack BH, Dart RC (2012). 2011 annual report of the American Association of Poison Control Centers’ National Poison data system (NPDS): 29th annual report. Clin. Toxicol.

[3] Athanassiadi K, Gerazounis M, Metaxas E, Kalantzi N (2002). Management of esophageal foreign bodies: a retrospective review of 400 cases. Eur J Cardiothorac Surg.

[4] Denney W, Ahmad N, Dillard B, Nowicki MJ (2012). Children will eat the strangest things: a 10-year retrospective analysis of foreign body and caustic ingestions from a single academic center. Pediatr Emerg Care.

[5] Silverman JA, Brown JC, Willis MM, Ebel BE (2013). Increase in pediatric magnet-related foreign bodies requiring emergency care. Ann Emerg Med.

[6] Hodges NL, Denny SA, Smith GA (2015). Rare-earth magnet ingestion–related injuries in the pediatric population: a review. Am J Lifestyle Med.

[7] Hussain SZ, Bousvaros A, Gilger M, Mamula P, Gupta S, Kramer R, Noel RA (2012). Management of ingested magnets in children. JPGN.

[8] Middelberg LK, Funk AR, Hays HL, McKenzie LB, Rudolph B, Spiller HA (2021). Magnet Injuries in Children: An Analysis of the National Poison Data System from 2008 to 2019. J. Pediatr.

[9] Abbas MI, Oliva-Hemker M, Choi J, Lustik M, Gilger MA, Noel RA, Schwarz K, Nylund CM (2013). Magnet Ingestions in Children Presenting to US Emergency Departments, 2002–2011. JPGN.

[10] Standards ASGE, of Practice Committee; Ikenberry SO, Jue TL, Anderson MA, Appalaneni V, Banerjee S, Ben-Menachem T, Decker GA, Fanelli RD, Fisher LR, Fukami N, Harrison ME, Jain R, Khan KM, Krinsky ML, Maple JT, Sharaf R, Strohmeyer L, Dominitz JA (2011). Management of ingested foreign bodies and food impactions. Gastrointest Endosc.

[11] Centers for Disease Control and Prevention (CDC) (2006). Gastrointestinal injuries from magnet ingestion in children--United States, 2003-2006. MMWR Morb Mortal Wkly Rep.

[12] Oestreich AE (2004). Multiple magnet ingestion alert. Radiology.

[13] Otjen JP, Rohrmann CA, Iyer RS (2013). Imaging pediatric magnet ingestion with surgical-pathological correlation. Pediatr Radiol.

[14] Horowitz R, Cico SJ, Bailitz J (2016). Point-of-care ultrasound: a new tool for the identification of gastric foreign bodies in children?. J Emerg Med.

[15] Hosokawa T, Yamada Y, Sato Y, Tanami Y, Nanbu R, Hagiwara SI, Oguma E (2016). Role of Sonography for Evaluation of Gastrointestinal Foreign Bodies. J Ultrasound Med.

[16] Kozaci N, Avci M, Pinarbasili T, Dönertaş E, Karaca A (2019). Ingested Foreign Body Imaging Using Point-of-Care Ultrasonography: A Case Series. Pediatr Emerg Care.

[17] Leibovich S, Doniger SJ (2015). The use of point-of-care ultrasound to evaluate for intestinal foreign bodies in the pediatric emergency department. Pediatr Emerg Care.

[18] Mori T, Nomura O, Hagiwara Y (2019). Another Useful Application of Point-of-Care Ultrasound: Detection of Esophageal Foreign Bodies in Pediatric Patients. Pediatr Emerg Care.

[19] Salmon M, Doniger SJ (2013). Ingested foreign bodies: a case series demonstrating a novel application of point-of-care ultrasonography in children. Pediatr Emerg Care.

[20] Carroll AG, Kavanagh RG, Ni Leidhin C, Cullinan NM, Lavelle LP, Malone DE (2017). Comparative Effectiveness of Imaging Modalities for the Diagnosis and Treatment of Intussusception: A Critically Appraised Topic. Acad Radiol.

[21] Dilley A, Wesson D, Munden M, Hicks J, Brandt M, Minifee P, Nuchtern J (2001). The impact of ultrasound examinations on the management of children with suspected appendicitis: a 3-year analysis. J Pediatr Surg.

[22] Navarro O, Daneman A (2004). Intussusception. Part 3: Diagnosis and management of those with an identifiable or predisposing cause and those that reduce spontaneously. Pediatr Radiol.

[23] Shen G, Wang J, Fei F, Mao M, Mei Z (2019). Bedside ultrasonography for acute appendicitis: An updated diagnostic meta-analysis. Int J Surg.

[24] Zhang H, Liao M, Chen J, Zhu D, Byanju S (2017). Ultrasound, computed tomography or magnetic resonance imaging - which is preferred for acute appendicitis in children?. A Meta-analysis. Pediatr Radiol.

[25] Shiu-Cheung Chan S, Russell M, Ho-Fung VM (2014). Not all radiopaque foreign bodies shadow on ultrasound: unexpected sonographic appearance of a radiopaque magnet. Ultrasound Q.

[26] Elissa M, Lubner MG, Pickhardt PJ (2020). Biopsy of Deep Pelvic and Abdominal Targets With Ultrasound Guidance: Efficacy of Compression. AJR Am J Roentgenol.

[27] Butterworth J, Feltis B (2007). Toy magnet ingestion in children: revising the algorithm. J Pediatr Surg.

